# Clinical Characterization of Patients with Chronic Spontaneous Urticaria according to Anti-TPO IgE Levels

**DOI:** 10.1155/2019/4202145

**Published:** 2019-12-07

**Authors:** Jorge Sánchez, Andres Sánchez, Ricardo Cardona

**Affiliations:** ^1^Group of Experimental and Clinical Allergy, Foundation “IPS Universitaria”, University of Antioquia, Medellín, Colombia; ^2^Foundation for the Development of Medical and Biological Sciences, Cartagena, Colombia; ^3^Immunology Department, University Corporation Rafael Núñez, Cartagena, Colombia

## Abstract

**Background:**

Chronic spontaneous urticaria (CSU) is a heterogeneous disease with some frequent comorbidities like autoimmune diseases, drug reactions, and inducible urticaria. IgE antibodies against thyroid peroxidase (anti-TPO IgE) could be associated with some of these clinical characteristics.

**Objective:**

To explore the clinical characteristics of CSU patients, according to the presence of anti-TPO IgE in serum.

**Methods:**

Anti-TPO IgE levels were measured during the clinical control period (Urticaria Activity Score, 0 point) and exacerbation period (≥3 points) in 100 CSU patients. Patients with self-reported exacerbation of skin involvement by foods, nonsteroidal anti-inflammatory drugs (NSAIDs), and physical triggers underwent controlled challenge tests.

**Results:**

We identified 2 groups of patients: (1) patients with anti-TPO IgE during the clinical control period or during an exacerbation, who had a higher frequency of atopy, asthma, and positive challenge test results with NSAIDs and (2) patients without anti-TPO IgE during any period, who had a higher frequency of positive challenge test results for inducible urticaria. Among the first group (anti-TPO IgE at any point), we identified 3 subgroups: patients with anti-TPO IgE during the clinical control period (*n* = 12); patients with anti-TPO IgE during the clinical control period and significantly increased levels during an urticaria exacerbation (*n* = 18); and patients with anti-TPO IgE only during an exacerbation (*n* = 13). None of the patients with self-reported food reactions had a positive challenge test result.

**Conclusion:**

Anti-TPO IgE is a useful biomarker for differentiating between clinical phenotypes of patients with CSU. Elevation of anti-TPO IgE during exacerbation periods supports an association between this autoantibody and the pathogenesis of urticaria.

## 1. Introduction

Chronic spontaneous urticaria (CSU) is a common cutaneous disease, affecting around 0.3% to 1% of the population worldwide [1]. Urticaria can appear at any time and has a marked impact on quality of life. Despite the spontaneous appearance of CSU, a subset of patients also associate the onset of symptoms with several triggers, namely, foods (10 to 40%), drugs (10 to 30%), and physical factors (35 to 70%), such as sports, sun exposure, tight clothing, and temperature (heat, cold) [2–4]. Consequently, patients may avoid certain activities and stop eating specific foods and taking drugs they need. These modifications in their activities of daily living have a considerable impact on the patient and do not necessarily lead to an improvement in symptoms, because the triggers are not always readily identified by the patient.

CSU is a heterogeneous disease with various clinical characteristics. Therefore, it could prove informative and is clinically important to identify biomarkers able to classify patients according to their phenotype based on underlying immunological mechanisms. Moreover, such an approach would enable us to stratify patients according to their clinical prognosis [5].

The association between thyroid autoimmunity and CSU has been evaluated in multiple studies [6, 7]. IgE antibodies against thyroid antigens such as thyroid peroxidase (anti-TPO IgE) are more frequent in CSU patients than in healthy individuals [8, 9]. Moreover, basophil activation by anti-TPO IgE suggests that this immunoglobulin could participate in exacerbations of urticaria [9], and *in vivo* results show that anti-TPO IgE can induce wheals in the skin of CSU patients [8]. Together, these results suggest a role for anti-TPO IgE in urticaria. Furthermore, evaluation of the clinical characteristics of patients with or without anti-TPO IgE can improve our understanding of the pathogenesis of the disease.

The aim of this study was to evaluate the clinical characteristics of CSU patients according to the presence of anti-TPO IgE in serum. We also investigated the role of anti-TPO IgE in exacerbations affecting the skin of these patients.

## 2. Methods

### 2.1. Study Population

The population assessed in this crosssectional study comprised patients recruited from the URTICA cohort (URTICA: Urticaria Research of Tropical Impact and Control Assessment) during the period 2017 to 2018 [10]. The CSU group included patients older than 12 years. This study was approved by the ethics committee of the clinic “IPS Universitaria” (Registration number IN40-2016).

Of the 155 patients with CSU who agreed to participate in the study, 55 were excluded owing to difficulties with serum sample collection during the exacerbation period; the final study population was 100 patients.

CSU was defined as the recurrence of hives, with or without angioedema, persisting for at least 6 weeks. The disease was diagnosed by an allergist or dermatologist. The exclusion criteria were systemic disease that could explain the hives and immunodeficiency, dermatitis, or any other disease that could alter the results of the serum tests. We also excluded pregnant women, patients with physical or mental disabilities, patients with decompensated cardiovascular disease, and patients with a chronic disease that could compromise challenge testing.

We used the Dermatology Life Quality Index to evaluate the impact on quality of life and the Urticaria Activity Score (UAS) to measure disease severity. The UAS was carried out for at least 7 consecutive days; if the patient present an UAS ≥ 3 points in at least one day, it was considered and exacerbation and serum sample was not recollected; if after 7 days it has not exacerbated, it was considered a clinical control period.

### 2.2. Study Design

The main objective was to evaluate the relationship between anti-TPO IgE and triggers of skin exacerbations in CSU such as NSAIDs, foods, and physical activity. As a secondary objective, we compared levels of anti-TPO IgE in periods of clinical control (UAS 0) or exacerbation of urticaria (UAS ≥ 3 points). Patients did not suspend the treatment with antihistamines that they had been receiving by their treating physician, so the periods of control and exacerbation occurred in the context of the clinical course. During exacerbation periods, the patient had hives and/or angioedema evidenced by the doctor at the same time the blood sample was taken. The urticaria triggers identified by patients were recorded in their clinical history. Possible inducible urticaria triggers, NSAIDs, and foods suspected by patients of being triggers of urticaria exacerbations were assessed directly using challenge tests.

Serum samples were collected from all patients during a period of clinical control and during 2 periods of exacerbations to determine anti-TPO IgE levels. At least two weeks were waited between the two exacerbation serum samples.

### 2.3. Challenge Tests

Patients taking daily second-generation antihistamines had to suspend their medication for at least 4 days before the challenge test. Any other drug that could have affected the outcome of the challenge test was suspended for the minimum time necessary before the challenge was performed. When patients had an exacerbation before the challenge test, a new appointment for a challenge test was offered.

According to the protocol, if patients had experienced a clear anaphylactic reaction within 1 hour of exposure to the trigger during at least 12 months before recruitment, the challenge test was avoided and considered positive. However, none of the patients met this criterion so we did not exclude any patient from the challenge tests. The protocols used for challenge tests were based on those proposed in international guidelines [11, 12], with modifications described elsewhere [2–4].

#### 2.3.1. Physical Challenge Test

The 5 most common self-reported sources of inducible urticaria (dermographism, cold, exercise, water, and pressure) [3] were evaluated in all the study patients, regardless of whether they were self-reported or not. Once the challenge tests were completed, the participants remained under observation for a period of 2 hours or more depending on the test performed.

#### 2.3.2. Challenge Test with Food

Oral challenge tests with food were blinded for the patient and placebo-controlled. The test was performed in patients with self-reported skin exacerbation of urticaria or angioedema. Patients received a portion equivalent to the expected daily intake of the food investigated. Blinding of food was done by mixing the food tested with other foods that the patient tolerated and that would help neutralize or at least decrease the taste of the food tested. Before the challenge test, specific IgE (sIgE) determination and skin prick testing (SPT) with the suspect food were performed with food extracts. In patients without atopy, challenge tests were performed by administering the total food serving in 2 portions separated by 1 hour (10% and 90% of the total serving, respectively). In patients with atopy, food was administered in 4 portions (10%, 20%, 30%, and 40%). The evaluation period after the challenge test was 4 hours, and the patients were instructed to notify the physician of delayed reactions. Delayed reactions that occurred in the first 24 hours after the provocation could be associated with the challenge; in that case, according to the protocol, we would repeat the challenge again to confirm the relationship.

#### 2.3.3. Challenge Test with NSAIDs

Oral challenge tests with NSAIDs were blinded for the patient and placebo-controlled. The test was performed in patients with self-reported skin exacerbation caused by NSAIDs. The equivalent to 1 daily dose of the drug was administered in 2 doses (10% and 90%) separated by 1 hour. In patients with a history of severe reactions (e.g., anaphylaxis or respiratory distress), the daily dose was administered in 4 steps (10%, 20%, 30%, and 40%) separated by periods of 1 hour. After the final dose was administered, the observation period at the clinic was 4 hours, and patients were also instructed to report any delayed reactions outside the clinic.

Some patients required a challenge test with 2 or more substances (e.g., a food and a medication). In patients with a history of reaction to 2 NSAIDs or 2 foods with crossreactivity, the first test was performed with the least probable trigger (e.g., meloxicam). The second trigger was tested only if the result with the first trigger was negative (e.g., ibuprofen).

When patients were discharged, they were advised that in the case of a late reaction, they should take a photograph and/or visit their health center. A challenge test was considered positive when the patient had hives or angioedema during the evaluation period. Other symptoms such as wheezing, diarrhea, and vomiting were also indicative of a positive test but were recorded separately if hives or angioedema were not present.

For challenge tests with food or medication, the placebo was administered at the beginning of the test. Subsequently, the physician performing the test could perform new administrations of placebo. The doctor performing the provocation was free to administer additional doses of placebo if, for example, he/she considered that the patient's pruritus could be due to anxiety or nerves of the test, always leaving an interval of at least 20 minutes with the next dose.

### 2.4. Levels of Anti-TPO IgE

Recombinant TPO was obtained as previously described [8]. Anti-TPO IgE was measured using ELISA. The results were expressed as the optical density (OD). The cut-off value (0.304 OD) for serum-specific IgE to TPO was defined as the mean and 3 times the standard deviation of absorbance values from 100 healthy controls without urticaria or autoimmune diseases [8]. Briefly, we made a calibration curve for each essay. Additionally, in each run, the samples were placed in duplicate, and a previously known positive serum control and a negative control (BSA) were used. Serum as the baseline and during exacerbation periods was taken in different days.

### 2.5. Statistical Analysis

The statistical analyses were performed using IBM SPSS Statistics for Windows, version 21.0 (IBM, Armonk, NY, USA). The mean was reported for descriptive variables. The Mann–Whitney test was used to compare anti-TPO IgE levels according to the cut-off. Pearson's *χ*^2^ test was used to evaluate differences between groups and proportions; in groups with a small number of events (≤5 events), Fisher's exact test was used. Correlations were assessed using the Spearman coefficient (*r*). We used the Kruskal Wallis test for quantitative variables (e.g., IgE levels); when this test revealed a significant difference, a multiple comparison test was performed to compare the differences between each possible pair of groups. A *p* value of ≤0.05 was considered statistically significant.

Based on the standard deviation observed in a control group of 100 healthy controls, in patients with positive anti-TPO IgE during the clinical control period, an increase in anti-TPO IgE levels during skin exacerbation was defined as significant if it was greater than 30% of the value recorded during the period of clinical control. In patients with negative anti-TPO IgE during the clinical control period, an increase was considered significant if the OD was greater than 0.304 during the exacerbation. Control subjects were patients over 18 years of age (29 confidence interval; CI 24–38) without previous or actual history of skin or autoimmune diseases, angioedema, or anaphylaxis.

Statistical analysis for multiple comparisons was done, for comparisons among more than two study groups; we use the Kruskal Wallis test, for quantitative variables (e.g., IgE levels).

## 3. Results

### 3.1. Population Characteristics

Most patients were female (Table 1). Forty-three patients had positive anti-TPO IgE during the clinical control period (UAS 0) or during at least 1 urticaria exacerbation (UAS ≥ 3). In these patients, total IgE, sensitization to mites IgE (atopy), and asthma were more frequent than those in patients with negative anti-TPO IgE at any time (Table 1).

**Figure 1:**
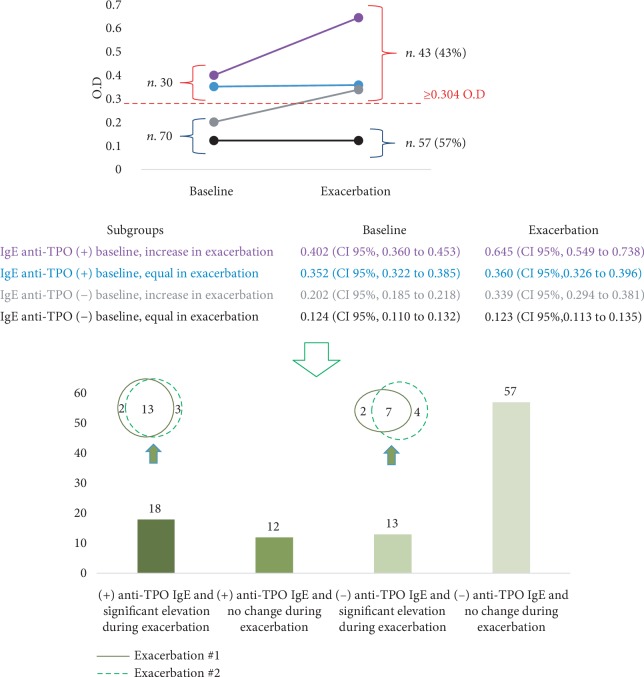
Groups of patients according to anti-TPO IgE level. Patients were evaluated during a period of clinical control (UAS 0) and two periods of urticaria exacerbation (UAS ≥ 3). Thirty patients had (+) anti-TPO IgE during the baseline period and 13 additional patients (*n* 43) during exacerbations (a). The number of patients according to the levels of anti-TPO in each period (baseline and two exacerbations periods) is presented in (b). At least two weeks was waited among the two exacerbation serum samples. OD: optical density.

Thirty patients had positive anti-TPO IgE during the clinical control period (Figure 1(a)). Eighteen (60%) had a significant increase in anti-TPO IgE during exacerbations (Figure 1(b)). Thirteen patients without anti-TPO IgE during the clinical control period presented a significant elevation of anti-TPO IgE in at least 1 of the 2 exacerbation periods. Fifty-seven patients without anti-TPO IgE during the clinical control periods experienced no significant change in this parameter during exacerbations (Figure 1(b)).

In the 43 patients with anti-TPO IgE elevation during the exacerbation of urticaria, we measured the levels of Der f, Blo t, and Can f; none of them had a significant increase in ration at baseline levels (*p* 0.18, 0.24, 0.12, respectively).

### 3.2. Inducible Urticaria according to Self-Reporting and Challenge Testing

**Figure 2:**
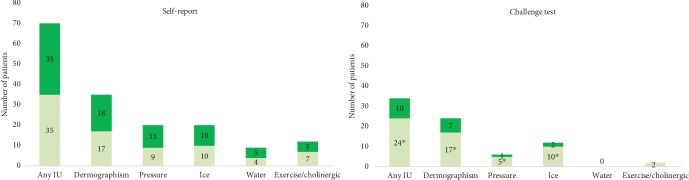
Prevalence of inducible urticaria according to self-reporting and challenge testing. Prevalence of inducible urticaria (IU) according to self-report and challenge test. Patients with (+) anti-TPO in dark green and (-) anti-TPO IgE in light green. ^∗^*p* < 0.05.

Seventy patients (70%) suspected at least 1 physical trigger, and 40 identified 2 or more. The most common self-reported trigger was dermographism (35%) (Figure 2(a)).

Thirty-four patients (34%) had a positive challenge test result (Figure 2(a)). The most frequent positive results were for symptomatic dermographism (24%), followed by cold (12%), pressure (6%), and exercise (2%). No patients had a positive challenge test result with water. According to the challenge test results, 10 patients (10%) had 2 different inducible urticaria.

Patients with negative anti-TPO IgE had a higher frequency of symptomatic dermographism, pressure urticaria, and cold urticaria than those in patients with positive anti-TPO IgE (Table 2). Symptoms during the challenge tests were mild and disappeared during the observation period. Adrenaline was not administered in any cases.

Additional physical challenge testing was performed in 4 patients whose exacerbations were strongly suspected of being caused by other triggers such as heat or sun exposure; the 4 challenges were negative (data not shown).

### 3.3. Evaluation of Foods as Triggers of Exacerbation of Urticaria

**Figure 3:**
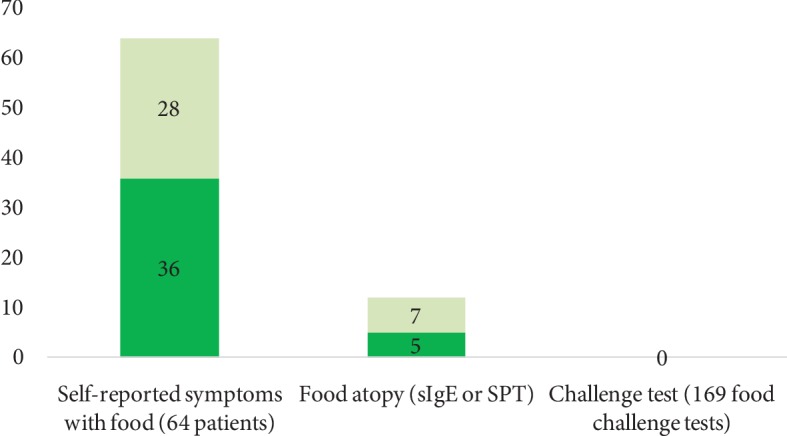
Prevalence of self-reporting, sensitization, and positive challenge test to foods. Food challenge tests were performed using the suspected food in patients who self-reported food allergy (*n* = 64). Each patient was challenged with the food or foods that he/she suspected of exacerbating urticaria (*n* 169). Patients with (+) anti-TPO in dark green and (-) anti-TPO IgE in light green. ^∗^*p* < 0.05.

Sixty-four (64%) patients reported a reaction with foods (Figure 3). Of these, 40 patients suspected 2 or more foods, and 56 (87.5%) had previous history of medical prescription of elimination diet. According to the self-report, multiple foods were suspected of causing the reaction: pork (*n* = 32), sauces (*n* = 30), spicy condiments (*n* = 20), egg (*n* = 19), milk (*n* = 20), sausage (*n* = 20), shrimp (*n* = 4), and fish (*n* = 4). Twenty other foods were reported by 1 or 2 patients. According to the SPT, 1 patient had IgE sensitization to pork, 2 to spicy condiments, 1 to milk, and 8 to shrimp.

We performed a total of 169 food challenge tests in 64 patients including those with a positive SPT result, although none were positive. During the administration of the placebo, 6 patients reported itching, but none presented objective reactions; therefore, the challenge test was continued and was tolerated in all cases, so there was not a positive challenge test with foods.

### 3.4. Evaluation of NSAIDs as Triggers of Exacerbation of Urticaria

**Figure 4:**
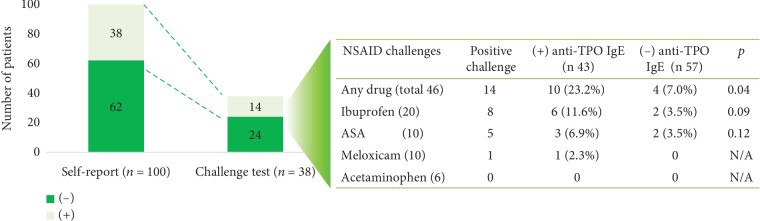
Comparison of NSAID self-report and challenge test results. A challenge test with the suspected NSAIDs was performed in patients who self-reported drug reaction. Each patient was challenged with the NSAIDs (≥1) that he/she had suspected of exacerbating the urticaria. N/A: not applicable; ASA: acetylsalicylic acid.

Thirty-eight patients reported at least 1 reaction with NSAIDs (Figure 4); of these, 18 (47%) reported reactions with 2 different NSAIDs. The reactions were reported with ibuprofen (*n* = 24), acetylsalicylic acid (ASA) (*n* = 18), meloxicam (*n* = 10), and acetaminophen (*n* = 6). A total of 56 NSAID challenge tests were indicated in 38 patients based on self-reports, although only 46 were performed: 4 patients refused to undergo the challenge, and 6 patients with self-reported reactions to 2 different NSAIDs had positive results in the first challenge test. Therefore, according to our study protocol, additional challenges with NSAIDs were rejected.

Fourteen patients had a positive challenge test result with NSAIDs; 2 required treatment with adrenaline. Nine of the 14 patients had a positive anti-TPO IgE level; this result was more frequent than in patients without anti-TPO IgE (Figure 4).

### 3.5. Clinical Characteristics of Patients with and without Anti-TPO IgE

**Figure 5:**
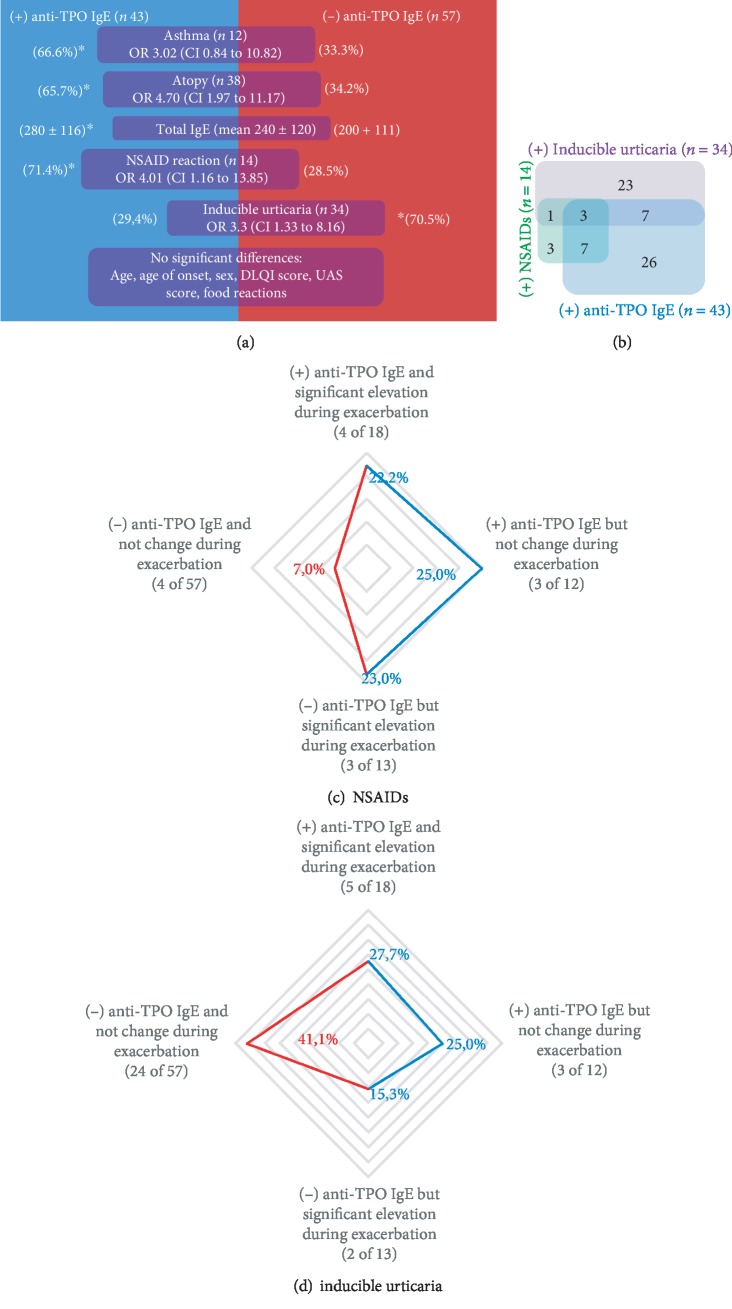
Anti-TPO IgE groups. The clinical characteristics of patients with or without anti-TPO IgE are represented in (a). (b) represents the interaction between patients with positive anti-TPO (blue), NSAID challenge testing (green), and inducible urticaria (purple). (c) and (d) present the frequency of patients with reactions to NSAIDs (*n* = 14) or inducible urticaria (*n* = 34) stratified by anti-TPO groups; with red line (-) anti-TPO group and in blue line (+) anti-TPO groups.

The clinical characteristics of patients with anti-TPO IgE differed from those of patients without anti-TPO IgE (Figures 5(a) and 5(b)). Patients with anti-TPO IgE (during control or exacerbation periods) had a significantly higher frequency of atopy, total IgE levels, and asthma; patients without anti-TPO IgE (during the control and exacerbation periods) had a higher frequency of inducible urticaria.

No statistically significant differences were observed when patients were stratified according to anti-TPO IgE levels during the clinical control or exacerbation periods (Figures 5(b) and 5(c), and supplementary material Tables [Supplementary-material supplementary-material-1] and [Supplementary-material supplementary-material-1]). Despite total IgE was higher in patients with anti-TPO IgE, correlation of total IgE and anti-TPO IgE levels was weak (*r* 0.403, *p* 0.18).

Autologous serum skin test (ASST) was more frequently positive in patients with positive anti-TPO IgE; nevertheless, it was not statistically different than patients with negative anti-TPO IgE (65% vs 58%, *p* 0.2). Among patients without anti-TPO IgE, we observed at the baseline, a tendency to have a greater number of eosinophils in the peripheral blood compared to the group with anti-TPO IgE (mean 173 CI 150-312 cells vs mean 142 120-260 cells); however, it was not statistically significant. We also found no differences with the number of basophils (CI mean 20 0-60 cells vs mean 18 5-55 cells). The frequency of thyroid disease and anti-TPO IgG was less than 15% in the groups. Statistical calculations were not performed as the number of subjects was not sufficient to make adequate comparisons.

## 4. Discussion

CSU is a heterogeneous disease with multiple clinical characteristics. Patients may have angioedema, inducible urticaria, reactions to NSAIDs, or autoimmune diseases. Biomarkers can improve our understanding of the underlying mechanisms of this disease [5, 13] and provide important information that enables us to classify patients according to different clinical phenotypes.

The presence of anti-TPO IgE may play a role in the development of CSU, as supported by data from *in vitro* and *in vivo* studies [8, 9]. We recorded production and elevation of anti-TPO IgE levels during skin exacerbations in patients with CSU; these results support the hypothesis that there is a relationship between anti-TPO IgE and urticaria. However, it was not always possible to detect the presence of this antibody during exacerbations, even in patients with anti-TPO IgE during periods of clinical control, suggesting that sometimes, the trigger can be these antibodies, but not always; other triggers (intrinsic and/or extrinsic) could explain the other exacerbations. These results do not demonstrate that anti-TPO IgE increases in a short-term fashion; if the increase in anti-TPO IgE during exacerbations was a progressive process or occurs abruptly was not explored in this study, since it would require a practically daily sampling of each patient's serum before the exacerbation to evaluate if there was a progressive or abrupt increase in IgE.

Several experimental studies support a relationship between IgG and IgE antibodies against self-antigens and urticaria; however, there are many questions that do not yet have answers but different studies support that these antibodies can activate and induce the release of histamine in the basophils of many patients with urticaria. In the case of anti-TPO IgE, it was recently demonstrated that passive transfer of serum from a patient with CSU and anti-TPO IgE to a healthy subject without CSU or anti-TPO IgE induced the formation of a wheal after TPO exposition [8]. This result, added to what has been observed in this study, supports the hypothesis that at least in a group of patients, IgE anti-TPO can be associated with the formation of wheals.

Previous studies (mostly with a retrospective design and small samples) have evaluated anti-TPO IgE as a possible prognostic biomarker of the duration and severity of CSU. However, evidence is inconsistent or weak [5, 13, 14]. It seems that these antibodies can help to identify 2 clinical phenotypes of patients with CSU: a phenotype associated with conditions linked to the Th2 response and reactions to NSAIDs, and a phenotype associated with the presence of inducible urticaria. This hypothesis is supported by indirect evidence: asthma, atopy, and high levels of total IgE are more common in CSU patients than those in healthy controls [8, 15–17]. In tropical urban areas, patients with a history of cutaneous reactions to NSAIDs had high levels of total IgE and mite-specific IgE [15, 16, 18], leukotrienes, and major genes of the IgE response (IL4, IL13), which are important in reactions to NSAIDs, as they are encoded at the same cluster in chromosome 5, suggesting a possible common expression [19, 20]. Shin et al. suggested that anti-TPO IgE plays a pathogenic role in aspirin-exacerbated cutaneous urticaria in terms of the expression of CD203c basophils [21]. The association of negative anti-TPO IgE group with inducible urticaria could be for different factors; as the calculation of the sample and the analysis of the outcomes were done by evaluating the presence of the TPO, the associations observed in the group without TPO can be a statistical artifact. However, we made corrections for analysis with multiple variables, and the association between negative anti-TPO and inducible urticarias (IUs) persists, which suggested a real association. Little is known about the mechanisms that occur in the different IUs; however, several hypotheses suggest non-IgE mechanisms (e.g., cholinergic mediation, terminal nerve receptors) [22].

Various underlying mechanisms may intervene in the development of CSU in patients without anti-TPO IgE, for example, IgG against Fc*ε*RI or IgE [23, 24], activation of the extrinsic coagulation pathway [25, 26], and IgE against autoantigens other than TPO [27]. It is not clear whether any of these mechanisms (or others) also play a role in inducible urticaria or whether they could facilitate its development. Independent of the underlying mechanism, the clinical characteristics of this group differ from those of the group with anti-TPO IgE, namely, lower levels of total IgE and lower frequency of atopy and reactions to NSAIDs.

We did not evaluate the therapeutic response of patients with CSU, although some studies suggest that high levels of total IgE have been associated with a better response to omalizumab [28–30]; in fact, one of these studies presents that patients with anti-TPO IgE have the highest complete respond rate in all clinical trials with omalizumab in CSU. According to these results, patients with anti-TPO IgE could be assumed to have a better clinical response, although we cannot assume the opposite (i.e., that patients without anti-TPO would have a diminished response), because this would depend on the underlying mechanism [31].

According to the levels of anti-TPO IgE during periods of clinical control and skin exacerbation, we identified 3 groups of patients with positive anti-TPO IgE. The responses according to the levels of anti-TPO IgE led us to ask whether these 3 groups were part of the same endotype at different time periods or whether they represented different mechanisms. The answer to this question may also help to clarify whether the presence of anti-TPO IgE in healthy subjects represents a risk for the development of urticaria. It is necessary to carry out prospective studies to resolve these issues.

Differences in the prevalence of inducible urticaria and drug reactions have been reported in different parts of the world [2–4, 15, 32–37], suggesting that in addition to the underlying mechanisms, other factors such as sociodemographic characteristics have to be considered as possible explanation for the discrepancies observed between the studies. Independent of the type of extrinsic trigger, we found that inducible urticaria and reactions to NSAIDs—but not to foods—are common comorbidities in patients with CSU and that self-reporting is usually sufficient to confirm associations. Therefore, challenge tests must be offered early during the medical evaluation to establish appropriate individualized avoidance measures and thus prevent unnecessary restrictions.

While our results are encouraging, our study is subject to limitations. Patients who initially had a value below 0.304 OD and during the exacerbation exceeded this value were considered positive since they exceeded the cut-off. However, in patients with a value close to the cut-off, this event could occur due to the expected variation between two tests (interassay variation of 10%) [8]. When we analyzed the increase of anti-TPO IgE during exacerbations in patients with negative IgE anti-TPO during the baseline, we observed that all of them had an increase greater than 30% in the values during an exacerbation, so it is unlikely that this increase is due to an error in the accuracy of the test. Given the study design and the age at recruitment, some clinical characteristics such as the frequency of autoimmune diseases or the severity of the disease could not be adequately evaluated between the anti-TPO IgE groups. Challenge tests with NSAIDs and foods were only performed in patients who self-reported their allergy; therefore, the number of reactions to NSAIDs may have been underestimated. However, from a clinical point of view, patients who do not have a history of NSAIDs or food reactions have no contraindications to any medication or food, with the result that it is not necessary for them to undergo challenge testing. While challenge testing is the gold standard for confirming the association between an exposure and the development of immediate symptoms, it does not ensure 100% tolerance to new exposures: the presence of cofactors (e.g., exercise, alcohol, infections) could affect the appearance (or nonappearance) of a reaction to a new exposure with the suspect substance [38–41]. Nevertheless, challenge testing is the most objective method of evaluating the reaction to an extrinsic trigger, and we performed these tests with NSAIDs, foods, and physical activities in all cases where testing was indicated; therefore, we consider that the clinical characterization of the groups was rigorous and robust.

In conclusion, anti-TPO IgE could be a useful biomarker for stratifying patients according to clinical phenotypes. Elevation of anti-TPO IgE during exacerbations supports a possible association between this autoantibody and the pathogenesis of urticaria which should be explored in more detail. This article presents new questions and new challenges that must be investigated. What could be driving the change in IgE anti-TPO levels? Which changes in IgG anti-TPO antibodies have to be addressed?

## Figures and Tables

**Table 1:** General characteristics. The general characteristics are presented in the 100 patients and in their two groups according to the presence or none of anti-TPO IgE. The “*p*” value was for the comparison between anti-TPO IgE groups ((+) anti-TPO IgE column and (-) anti-TPO IgE column). ^∗^Atopy to mites and/or pets. DLQI: Dermatology Life Quality Index; UAS: Urticaria Activity Score; N/A: not applicable. ± standard deviation. Ns: not significant.

Variables	(+) anti-TPO IgE (*n* = 43)	(-) anti-TPO IgE (*n* = 57)	*p*
Age	30 (16 to 48)	29 (16 to 50)	Ns
Age at onset (y)	20 (6 to 48)	23 (6 to 48)	Ns
Sex: female, *n* (%)	28 (65.1%)	35 (61.4%)	Ns
Total IgE (IU/ml)	280 ± 116	200 ± 111	0.04
Atopy, *n* (%)^∗^	25 (58.1%)	13 (22.8%)	0.04
Asthma, *n* (%)	8 (18.6%)	4 (7%)	0.05
Rhinitis, *n* (%)	19 (44.1%)	21 (36.8%)	Ns
Autoimmune diseases	9 (20.9%)	9 (15.7%)	Ns
DLQI score, mean ± SD	18 ± 3	16 ± 4	Ns
UAS score, mean ± SD	3 ± 2	3 ± 2	Ns

**Table 2:** Inducible urticaria according to challenge testing and anti-TPO IgE. Comparison of patients with or without anti-TPO IgE according inducible urticaria.

Inducible urticaria according to challenge testing	(+) anti-TPO IgE (*n* = 43)	(-) anti-TPO IgE (*n* = 57)	*p*
Patients with any inducible urticaria	10 (23.2%)	24 (42.1%)	0.03
Dermographism	7 (16.2%)	17 (29.8%)	0.03
Pressure	1 (2.3%)	5 (8.7%)	0.04
Ice	2 (4.6%)	10 (17.5%)	0.02
Water	0	0	N/A
Exercise/cholinergic	0	2	N/A

## Data Availability

This study was based on data from the “URTICA” cohort.

## Abbreviations

CSU: Chronic spontaneous urticaria
Anti-TPO IgE: IgE antibodies against thyroid peroxidase
IU: Inducible urticaria
NSAIDs: Nonsteroidal anti-inflammatory drugs
OD: Optical density
UAS: Urticaria Activity Score.

## Supplementary Materials

Supplementary Materials Supplementary Table 1: physical challenge tests stratified by anti-TPO groups. Comparison of anti-TPO IgE groups. Group 1: patients with anti-TPO IgE during the clinical control period and significantly increased levels during an exacerbation (*n* = 18). Group 2: patients with anti-TPO IgE during the clinical control period with no increase during an exacerbation (*n* = 12). Group 3: patients with anti-TPO IgE only during an exacerbation (*n* = 13). N/A, not applicable. Supplementary Table 2: NSAID challenge tests stratified by anti-TPO groups. *p* value for anti-TPO IgE groups. Group 1: patients with anti-TPO IgE during the clinical control period and significantly increased levels during an exacerbation (*n* = 18). Group 2: patients with anti-TPO IgE during the clinical control period with no increase during an exacerbation (*n* = 12). Group 3: patients with anti-TPO IgE only during an exacerbation (*n* = 13). N/A, not applicable. ASA, acetylsalicylic acid.
