# Supplementary material for: Clinical Characterization of Patients with Chronic Spontaneous Urticaria according to Anti-TPO IgE Levels
Source: J Immunol Res. 2019 Dec 7;2019:4202145. doi: 10.1155/2019/4202145 (PMC6925798; doi:10.1155/2019/4202145)
Supplement: Supplementary Materials — Supplementary Table 1: physical challenge tests stratified by anti-TPO groups. Comparison of anti-TPO IgE groups. Group 1: patients with anti-TPO IgE during the clinical control period and significantly increased levels during an exacerbation (n = 18). Group 2: patients with anti-TPO IgE during the clinical control period with no increase during an exacerbation (n = 12). Group 3: patients with anti-TPO IgE only during an exacerbation (n = 13). N/A, not applicable. Supplementary Table 2: NSAID challenge tests stratified by anti-TPO groups. p value for anti-TPO IgE groups. Group 1: patients with anti-TPO IgE during the clinical control period and significantly increased levels during an exacerbation (n = 18). Group 2: patients with anti-TPO IgE during the clinical control period with no increase during an exacerbation (n = 12). Group 3: patients with anti-TPO IgE only during an exacerbation (n = 13). N/A, not applicable. ASA, acetylsalicylic acid. [file 4202145.f1.pdf]

Supplementary material.

Supplementary table 1.

Inducible urticaria according to challenge test	(+) Anti-TPO IgE (n 43)	Group 1 (n 18)	Group 2 (n 12)	Group 3 (n 13)	*p*
Patients with any inducible urticaria	10 (23.2%)	5	3	2	0.14
Dermographism	7 (16.2%)	4	2	1	0.13
Pressure	1 (2.3%)	1	0	0	N/A
Ice	2 (4.6%)	1	1	0	N/A
Water	0	0	0	0	N/A
Excercise//cholinergic	0	0	0	0	N/A

Supplementary Table 1. Physical challenge tests stratified by anti-TPO groups. Comparison of anti-TPO IgE groups. ***Group 1***: patients with anti-TPO IgE during the clinical control period and significantly increased levels during an exacerbation (n=18). ***Group 2***: patients with anti-TPO IgE during the clinical control period with no increase during an exacerbation (n= 12) ***Group 3***: patients with anti-TPO IgE only during an exacerbation (n= 13). N/A, not applicable.

Supplementary table 2.

NSAID challenge	Positive challenge	(+) Anti-TPO IgE (n 43)	Group 1 (n 18)	Group 2 (n 12)	Group 3 (n 13)	P
Any NSAID (total 46)	14	10 (23.2%)	4	3	3	0.14
Ibuprofen (20)	8	6 (11.6%)	2	2	2	0.15
ASA (10)	5	3 (6.9%)	1	1	1	0.14
Meloxicam (10)	1	1 (2.3%)	1	0	0	N/A
Acetaminophen (6)	0	0	0	0	0	N/A

Supplementary Table 2. NSAID challenge tests stratified by anti-TPO groups. *P* value for anti-TPO IgE groups. ***Group 1***: patients with anti-TPO IgE during the clinical control period and significantly increased levels during an exacerbation (n=18). ***Group 2***: patients with anti-TPO IgE during the clinical control period with no increase during an exacerbation (n=12) ***Group 3***: patients with anti-TPO IgE only during an exacerbation (n=13). N/A, not applicable. ASA, acetylsalicylic acid.
